# Lectures on Clinical Surgery

**Published:** 1855-05

**Authors:** James Syme

**Affiliations:** Professor of Clinical Surgery in the University of Edinburgh


LECTURES ON CLINICAL SURGERY. By JAMES SYME, ESQ., Professor of Clinical Surgery in the University of Edinburgh.

LECTURE IV. *Necrosis and Caries.*—When this man (S. R., aged 40,) was admitted to the Infirmary, ten weeks ago, his right foot presented a great degree of swelling; the parts were matted together, and there was a profuse discharge from several openings on the dorsal and plantar aspects. This state of things had resulted from injury sustained nine weeks previously by his treading upon a sharp hook, which lay with the points presenting upwards. The appearance of the foot was formidable, and the patient came here expecting that the removal of part or the whole of it would be necessary; whereas he has been relieved on much easier terms. The foot is now sound, and he is able to walk upon it. This case is interesting on its own account, and also on account of some important principles of surgical practice involved in it. What was the essential obstacle to healing? There could be no doubt it was seated in the bone, just as in disease of the elbow-joint, where there may be a great degree of swelling, and other appearances which might suggest to an experienced eye the idea that the soft parts were those chiefly concerned, yet soon after the removal of the diseased portions of bone, the soft parts acquire a healthy appearance.

The diseased bone might be either an exfoliation lying imprisoned amongst dense textures not admitting of its escape, or it might be in the state of caries. With reference to the question, which of these two conditions was present, we must particularly consider the nature of caries. Caries is often spoken of as equivalent to an ulcer of the bone, but this is not correct, for you may induce an ulcer in bone, which will heal readily—*e. g.*, the spongy bone which is divided in excision of the elbow-joint assumes afterwards the same condition as an ulcer of the soft parts, yet it heals without any remarkable obstinacy, which is the chief characteristic of caries. When a joint is opened by a wound, if union by first intention do not occur, inflammation with suppuration necessarily follows, but caries may or may not result. A gentleman of my acquaintance once removed a loose cartilage from the knee of a patient. Inflammation of the joint came on, with suppuration, which was very profuse when I saw the case, but recognising some points about it that led me to think favorably, I recommended longer delay, and the discharge ultimately dried up. In one of the cases in which you saw excision of the elbow-joint performed, there was anchylosis resulting from injury, the articular cartilages had been removed during the suppurating process, yet the osseous surfaces were firmly united, which they could not have been if caries had existed between them. Pressure, it is said, will cause caries, but this is a great error; tumors pressing on bones may cause interstitial absorption—*e. g.*, an atheromatous tumor, pressing upon the frontal bone, may produce a depression on its surface, which however still remains covered by its periosteum; still further, the pressure of an aneurism may cause the absorption of large portions of the vertebræ, or of the whole thickness of the sternum, through which the tumor may protrude beneath the integument, or again of half the clavicle. This is not caries however; and no one can doubt, that if the aneurism were cured, the bone would heal. It is said, that pressure produced by matter causes caries; but this is a very erroneous and mischievous opinion. When matter is formed in the interior of a bone, as, for instance, in connexion with an exfoliation, the pressure of the matter causes openings in the surrounding bone; but no one supposes that the edges of these openings are carious. How, then, is the caries produced? Inflammation, it would seem must necessarily precede it, and it depends upon the condition of the part, or the constitution of the patient, whether caries will follow or not. This, you will say, is very unsatisfactory; but it is as far as we can go at present. And I beg to remind you, that there is no more certrin step towards the attainment of truth, than becoming conscious of our own ignorance. This is a dark point in pathology, which it is not impossible some gentleman now before me may be destined to clear up; and, meanwhile, all that we can do as practical men is to consider the circumstances that favor the production of caries. In the first place, the age of the patient appears to have great influence in determining the result of inflammation of bone; the younger the patient, the less is the risk of inflammation followed by suppuration going on to caries; for I need scarcely say, that if there be no suppuration, there is no caries. Hence the success of certain operations which have been performed on young patients almost infants, which, if tried on older persons, would probably have met with less satisfactory results; the older the patient, the greater is the risk of caries, and the chances of its occurrence are also greater the more unsound the constitution, or, as is generally said, the more scrofulous the constitution. The term scrofula in generally understood to imply a specific tendency depending upon hereditary taint; but the expression weak or unsound constitution, is probably, in reality, nearer the truth. Give me the strongest child, and I could undertake to make him scrofulous by bringing him up in a damp cellar, with deficient food, and imperfect clothing. No doubt this state of constitution may be transmitted from the parent; but it may also as certainly be acquired. If the suppuration is the result of a wound, the danger of caries is much smaller than if it has arisen from some injury other than a wound—such as a twist or sprain. It is impossible for you to be too much impressed with this fact. More than twenty years ago a young man, employed by a butcher at Leith, was standing on a bench putting a joint of meat on a hook above him, when the bench slipped, and, as he fell the hook pierced his wrist, entering at the palmar aspect, and passing obliquely through the carpus almost to the back of the hand. He was afterwards sent to Minto House, which was then occupied as a surgical hospital under my care. There was a great degree of inflammation about the wrist; but being at that period aware of the distinction which I am pointing out to you, I said, that as the suppuration was the result of a wound, it was right to give the limb a chance. Free vent was, therefore, given to the matter by incision, but no improvement followed; and, at the end of six weeks, the discharge was profuse, and grating was felt when the bones were moved. Amputation was now proposed; but the patient expressed a wish to go home for a short time, and try, as a last resource, the effect of change of air. He did so, and returned before many weeks were over with his hand perfectly well.

I might mention many cases, but will content myself with two others that appeared to me peculiarly illustrative. A groom ran a penknife accidentally into his wrist, inflammation and suppuration followed, and he was brought to this infirmary. Within a day or two, a female patient was admitted, with swelling of the wrist and fluctuation, resulting from a sprain. In speaking of these two cases in this theatre, I expressed the opinion that in the case of the man, as the derangement had resulted from a wound, it might perhaps be cured; but that the woman's wrist, although less swollen, having had a different origin, would probably prove incurable. Both were treated alike, free vent was afforded to the matter, both were placed in splints, and both dressed with a lotion.

For awhile, the only difference between the two cases was, that the appearance of the man's wrist was much the more formidable; but, after a time, improvement began in him, and he went on to a perfect cure. But, in the woman's case, after the disease had remained apparently stationary for a while, the pain increased, the discharge became more profuse, her health began to suffer, and at length we acceded to her request, and amputated the limb. It is peculiarly incumbent on young practitioners to wait and allow time for the case itself to declare what its issue will be. If the disease has originated in a wound, and the patient is young and of good constitution, you will no doubt think more favorably of the case than if the opposite conditions were present; but I would advise you to be slow to act upon any of these modes of guidance, and to trust to time to decide for you; while the treatment which you will adopt, in the meantime, will be to allow free drain to the matter, to keep the limb, as much as possible at rest, and to attend to the general health. In the patient before you, the disease, as I before mentioned, had originated from a wound, a hook having pierced the foot, and caused inflammation and suppuration. On examination with the probe, I found that the disease, instead of affecting all the bones of the tarsus, was confined to the joint between the second toe and its metatarsal bone; and it further appeared that some small exfoliations lay loose in the middle of the foot. I removed these, and they proved to be, as you see, thin shells of dense bone; one of them, which is convex, corresponding to the rounded end of the metatarsal bone, and the other, to the concave articular surface of the phalangæal bone. The dense bone had died from the inflammation excited by the injury, while the loose osseous texture adjacent had ulcerated sufficiently to throw off the dead portions, but was not carious, for, since the removal of the exfoliations, the swelling has gradually diminished, the discharge has almost ceased, and he can bear his weight upon the foot, and recovery may be said to be complete.

There is another case to which I will now call your attention, as the same principles are concerned in it; it is one of sinuses about the pelvis, presenting some similarity to that of T. C——, from whom I lately removed an exfoliation of the rami of the ischium and pubes. The patient is J. U——, aged twenty-five, obviously of a very weak and unhealthy constitution, employed in a silk mill at Congleton, in Cheshire, where he was sometimes engaged for three hours together in lifting from the floor to the level of his head the wooden rollers used in silk-carding. Two weeks after last Christmas (1853) he began to suffer great pain in the region of the tuberosity of the left ischium, and a small lump about as large as a pea appeared on the back of the thigh, an inch or two below that prominence. The lump increased in size and the pain became so severe, that he was obliged to give up work. Poultices were ordered by his medical attendant, and soon after their application the tumor broke, and discharged a large quantity of matter; the surgeon also lanced another place near the first, and a third opening subsequently formed lower down on the back of the thigh. A profuse discharge has continued ever since from these apertures, and he has become much emaciated. He was recommended to seek my advice, and came to Edinburgh accordingly, and was admitted to the infirmary on the 25th of August. At this time I enlarged one of the sinuses sufficiently to admit my finger, and found that it led up towards the pelvis, but could not discover any exfoliation. He continued to lie here without much change for two months, when I determined to explore the sinuses still more thoroughly; and he was brought into this theatre, as you will remember, and chloroform having been administered, I enlarged the highest opening freely with a probe-pointed bistoury, and on introducing my finger, discovered a large part of the surface of the tuberosity of the ischium bare and rough, but firmly fixed. Considering the length of time that the disease had existed, it appeared to be probable that it was caries; but I thought it best to let the patient lie quiet for a few weeks, when if there were an exfoliation, it would be either loose or so far detached that we should be able to recognise it as such. Four weeks have elapsed, and I propose to examine the man again now; but before he comes, let us consider the course we must take if, as I expect, we find the bone carious. He is losing flesh and strength. Can anything be done for him if the disease is of that incurable kind which we call caries? If it is limited to the tuberosity of the ischium, I see no objection to cutting out that part. It may e said that, so far as I know, no one has ever yet performed such an operation, but that is no reason at all why we should not; if we are satisfied that the operation is expedient and practicable, the mere absence of precedent is no obstacle. The tuberosity of the ischium may be reached without risk of injury to any important parts, and the mere division of the attachment of the muscles is of no moment, for we know that, as after excision of the elbow-joint, the muscles will acquire fresh attachments. The operation is therefore practicable, and it is also expedient, for we can make sure of removing the whole of the disease. In this respect the present case is very different from those in which the hip-joint is the part affected; there it is of no use to attempt removal of the disease, for it cannot be completely taken away, since the acetabulum is not only affected, but is generally more diseased than the head of the femur. You will see persons in the streets with one leg shorter than the other, and the toes everted or inverted, as the case may be, moving on with a greater or less degree of lameness; these are people who have had inflammation and suppuration of the hip-joint, so that recovery is no proof that the operation was necessary or expedient. I incline to attribute the recovery in these cases to the fact that the patients have generally been young, and taken from the poorest classes, and have had every attention lavished upon them, wine and cordials, and delicate articles of food being freely given them, and then, in their convalescence, they have been sent to Margate or Brighton, or elsewhere, for change of air. It may, perhaps, still be said that cutting out the head of the femur may promote recovery, and make it more complete; I say, that if anchylosis occurs, the limb remains more or less useful; but if you cut out the head of the bone, it can never be of any good as a support to the body; it will be just as immediately after fracture of the neck of the femur, or as if the head of the bone were congenitally absent; the patient may carry the limb, but the limb can never carry him.

[The man was now brought into the theatre, chloroform was given, and Mr. Syme, on repeating the exploration of the sinus with his finger, found the diseased bone still firmly fixed, and with the same bare, rough surface as before. Mr. Syme mentioned these facts to the class, and expressed his intention of removing the disease. Next day the patient was again put under chloroform, and turned round upon his face; Mr. Syme then inserted a curved, probe-pointed bistoury into the large sinus situated at the inner part of the natal fold, and cut freely upwards through the mass of the buttock, so as to expose the posterior surface of the tuberosity of the ischium completely. Finding that the disease did not involve the whole tuberosity, but was limited to its posterior aspect, he removed the carious parts with the bone-pliers till nothing but perfectly sound bone remained. Two small vessels alone required ligature. A piece of lint was placed on the wound, and the patient taken back to bed. On examination of the piece of bone removed, the diseased part presented the ordinary appearance of caries, which involved the greater part of the posterior surface of the tuber ischii, and penetrated rather more than half an inch into its substance.

On the following day, Mr. Syme, in speaking of the case in his lecture, pointed out the similarity between this man's employment and that of the first patient, from whom he removed an exfoliation of the pelvis, who, just before the commencement of his symptoms, had been engaged in curing herrings, alternately stooping down to lift them from the ground, and stretching up to the full extent to place them above the level of his head. In that case the extraordinary strain upon the flexor muscles had caused inflammation at their origin, which had led to an exfoliation from the tuberosity of the ischium; in the present case, the patient, as will be remembered, was frequently engaged for three hours together in taking up silk carding rollers from the floor and placing them at the level of his head, but the inflammation excited in the tuberosity of the ischium had in this case ended in caries; the difference between the results in the two patients probably depending on difference of constitution. No bad consequences followed the operation, and the discharge, which had previously been very profuse, became proportioned to the wound in the soft parts, which has been steadily contracting.]

---

LECTURE V. *Fracture of the Skull.*—This young man, John W——, aged twenty-three, was at work at a flour-mill in Kirkaldy on the 25th of July last, when a fourteen pound weight fell from the fourth story, and struck him on the head, about an inch and a half from the vertex, where you now observe a depressed cicatrix. On his admission here, I found a depressed fracture of limited extent, and, on removing with bone-pliers the fragments of the outer table, discovered a circular portion of the inner table, of much larger size, which I succeeded in extracting piecemeal through the opening in the outer table. He was conscious at the time, and has ever since been in the full enjoyment of his intellectual faculties. The wound was simply dressed, and went on favorably till its contraction produced inversion of the edges of the scalp, when the hairs growing into the wound prevented further cicatrization, till I pared away the inverted edge a few weeks ago, after which the wound soon healed completely.

In such cases as this there is no reproduction of bone in the central part of the cicatrix, which is intimately adherent to the dura mater; and though it becomes after a time exceedingly dense, yet it is prudent for the patient to wear some sort of protection if liable to be exposed to external violence.

I may take this opportunity, gentlemen, of remarking that injuries of the head have long been a fruitful subject of bad practice and traditional error. In the olden times injury of the head, accompanied with insensibility, was thought to require scalping of the part, so as to allow a thorough examination of the bone, when if a fissure was discovered, the trephine was applied, under the idea that noxious transudations were liable to pass from without inwards. Even in cases where there was no evidence of cerebral disorder, it was the practice to remove any flap of the scalp that might have been torn up from the cranium. So lately as the time of Charles II., we find it mentioned in the valuable cases recorded by Wiseman, then sergeant-surgeon, that a surgeon who had cut off a large flap of skin in these circumstances was so proud of his performance, that he hung up the piece of scalp in his shop, to show what a great operator he was. Even in recent times, whenever injury of the head produced loss of consciousness, it was the practice to bleed largely immediately after the accident.

All these errors have now been disposed of, but something still remains to be cleared away. You have, no doubt, all of you heard of the operation of trephining, and have read that it is required under the following circumstances:—1st, When purulent fluid exists between the bone and the dura mater; 2nd. When blood has been effused from the main artery of the dura mater; 3rd. When the bone is depressed injuriously: and you will perhaps be somewhat surprised when I tell you that, if you except the case of punctured fracture of very small size, such as a sharp-pointed instrument would produce, trephining is never of any service. Take first the cases of effusion of matter and of blood. Though I have been now nearly thirty-five years connected, in one way or other, with this hospital, I have never known a single case where blood or matter has been withdrawn with a satisfactory result; indeed, I have never seen blood withdrawn at all by trephining, nor did I ever find a surgeon who had met with such a case. You will therefore do well to regard trephining under these circumstances as an operation that does not at all concern you. Also in depressed fracture, if you except the special case of small puncture, you will find trephining never necessary. If the bone is broken, it is so much so that it can be elevated by bone-pliers. Make a crucial incision in the scalp, and raise the flaps; try different parts with the bone-pliers till you can raise a small portion; cut off bits of the overhanging edge, if necessary, and you will be able to complete the elevation of the whole. If the bone has not injured the dura mater, the patients generally recover; if the longitudinal sinus is injured, they die, so far as my experience goes, although there appears no special reason why injury of this venous channel should be so much more serious than that of others, and cases of recovery have been recorded. If the dura mater has been injured so as to expose the brain, the case is very unfavorable; and if the brain itself is injured, it is almost hopeless, I say, *almost* hopeless, for it is not entirely so. A little more than a year ago a fire occurred in the Cowgate, which, as you know, is an old street of lofty houses. The occupiers of the fourth story threw out their valuables into the street, and, amongst the rest, a child four years old. It appeared to have fallen on its head, and had injured one parietal bone, over which a soft tumor existed. One day passed, and next day it appeared that some epileptic seizures had occurred, which, on the following day, became almost continuous, the child being scarcely out of one fit before another came on. I thought it right to open the tumor, and on my doing so, blood issued, and a wide crack appeared in the parietal bone, which had given exit to a quantity of cerebral substance, the appearance altogether being so frightful that I did not complete the incision, but regarding the case as hopeless, sent the child to bed with a piece of wet lint on the wound. However, the convulsive attacks ceased from that time forward; the child showed signs of intelligence within two hours; on the third day it was sitting up, with a smile on its countenance; and in a fortnight after the operation it was running about the ward. I learned a short time since that this child was in the most exuberant health. But such cases are unfortunately quite exceptional.

I may remark, as we have happened to speak on this subject, that epileptic attacks sometimes come on months or years after injuries of the head; and you may perhaps be called upon to "do something for the patient," as it is said—that is to say, to trephine the skull. I have myself on several occasions exposed the bone and removed portions in such cases; but, from the results which have followed, I advise you positively against such a course. The The case is different, however, if a sinus continue in connexion with a sharp piece of bone pressing on the brain. I once operated on such a case, and some years afterwards had the satisfaction of seeing the patient in perfect health. The case is thus reported in the Tenth Report of the Edinburgh Surgical Hospital for 1833, (*Edinburgh Medical and Surgical Journal*, No. 115.)

Wm. K——, aged twenty-two, from Dunfermline, was admitted on the 3d of September. Between six and seven years ago he suffered a very severe wound of the face from the blasting of a rock. He was at first insensible, and considered in a hopeless state, but gradually recovered so as to be able to follow his employment as a weaver. A small opening, however, between the cheek-bone and ear continued to discharge matter, and he occasionally suffered much pain in his head. During the last four months he had had occasional epileptic fits. On examination, it appeared that the malar bone had been fractured, and the whole upper part of the face was occupied by a dense cicatrix. A piece of bone was felt at the bottom of the sinus, bare, and lying obliquely in respect to the surface of the skull. On the supposition that this was a portion of the cranium which had been depressed at the time of the injury, a crucial incision was made through the cicatrix, to expose the aperture of the skull, which was then enlarged by means of cutting-forceps, so as to admit the extraction of the loose piece. It comprehended both tables of the bone, (temporal,) and was about a third the size of a sixpence. The patient expressed much relief after the operation; but the fits still recurred, though seldom, and a profuse discharge issued from the wound. A probe being gently introduced, entered to the depth of two inches perpendicularly, the substance of the brain seeming to have been hollowed out into an abscess. He returned home, and continues in the same state, though, on the whole, improved.—*London Lancet.*

